# Involvement of JNK-mediated pathway in EGF-mediated protection against paclitaxel-induced apoptosis in SiHa human cervical cancer cells

**DOI:** 10.1054/bjoc.2001.1910

**Published:** 2001-07

**Authors:** B Liu, M Fang, Y Lu, Y Lu, G B Mills, Z Fan

**Affiliations:** Department of Experimental Therapeutics, Department of Molecular Therapeutics, The University of Texas M. D. Anderson Cancer Center, Houston, Texas, 77030, USA

**Keywords:** paclitaxel, apoptosis, EGF, JNK, PI-3K, MAPK

## Abstract

We investigated the signalling pathways by which epidermal growth factor (EGF) modulates paclitaxel-induced apoptosis in SiHa human cervical cancer cells. SiHa cells exposed to paclitaxel underwent apoptosis, which was strongly inhibited by EGF. This inhibition of apoptosis by EGF was not altered by pharmacological blockade of phosphatidylinositol 3′-OH kinase (PI-3K) with the PI-3K specific inhibitor LY294002 or blockade of the mitogen-activated protein kinase (MAPK) kinase (MEK) with the MEK specific inhibitor PD98059, or by transfection of the cells with PI-3K or MEK dominant-negative expression vectors. EGF did not stimulate PI-3K/Akt, MEK/MAPK, or p38 MAPK activity in SiHa cells but did transiently activate the c-Jun NH2-terminal kinase (JNK). Co-exposure of SiHa cells to SB202190 at concentrations that inhibit JNK abolished the protective effect of EGF on SiHa cells against paclitaxel-induced apoptosis. Our findings indicate that the JNK signaling pathway plays an important role in EGF-mediated protection from paclitaxel-induced apoptosis in SiHa cells. © 2001 Cancer Research Campaign http://www.bjcancer.com


*British Journal of Cancer* (2001) **85**(2), 303–311
© 2001 Cancer Research Campaign
doi: 10.1054/ bjoc.2001.1910, available online at http://www.idealibrary.com on IDEAL®

http://www.bjcancer.com

### Involvement of JNK-mediated pathway in EGF-mediated protection against paclitaxel-induced apoptosis in SiHa human cervical cancer cells

**B Liu[1], M Fang[1], Y Lu[1], Y Lu[2], GB Mills[2] and Z Fan[1,2]**

[1]Department of Experimental Therapeutics and [2]Department of Molecular Therapeutics, The University of Texas M. D. Anderson Cancer Center, Houston, Texas 77030, USA

**Summary** We investigated the signalling pathways by which epidermal growth factor (EGF) modulates paclitaxel-induced apoptosis in SiHa human cervical cancer cells. SiHa cells exposed to paclitaxel underwent apoptosis, which was strongly inhibited by EGF. This inhibition of apoptosis by EGF was not altered by pharmacological blockade of phosphatidylinositol 3′-OH kinase (PI-3K) with the PI-3K specific inhibitor LY294002 or blockade of the mitogen-activated protein kinase (MAPK) kinase (MEK) with the MEK specific inhibitor PD98059, or by transfection of the cells with PI-3K or MEK dominant-negative expression vectors. EGF did not stimulate PI-3K/Akt, MEK/MAPK, or p38 MAPK activity in SiHa cells but did transiently activate the c-Jun NH2-terminal kinase (JNK). Co-exposure of SiHa cells to SB202190 at concentrations that inhibit JNK abolished the protective effect of EGF on SiHa cells against paclitaxel-induced apoptosis. Our findings indicate that the JNK signaling pathway plays an important role in EGF-mediated protection from paclitaxel-induced apoptosis in SiHa cells. © 2001 Cancer Research Campaign http://www.bjcancer.com

**Keywords**: paclitaxel; apoptosis; EGF; JNK; PI-3K; MAPK

The epidermal growth factor (EGF) is one of the most important mitogens for many epithelial cells and typically promotes cell proliferation through the well-characterized Grb2/SOS/Ras/Raf/ERK pathway (Ullrich and Schlessinger, 1990). Recent studies from several laboratories have shown that EGF can also act as a survival factor in suppressing apoptosis induced by various death signals (Caraglia et al, 1999; Gibson et al, 1999; Lan and Wong, 1999; McClellan et al, 1999; Payne et al, 1999; Leu et al, 2000). This latter function is performed primarily by EGF receptor-mediated activation of the phosphatidylinositol 3′-OH kinase (PI-3K) pathway and/or the mitogen-activated protein kinase (MAPK) pathway. EGF activates PI-3K through EGF receptor-associated substrate molecules, such as the Grb2-associated binder-1 (Gab1), that form the docking sites for the SH2 domains of the p85 adapter subunit of PI-3K. This recruits PI-3K to proximity with the EGF receptor, enabling subsequent phosphorylation and activation of PI-3K (Rodrigues et al, 2000). Additionally, EGF can also activate PI-3K through the small guanosine triphosphatase (GTP)-binding protein Ras, which interacts directly with the catalytic subunit of PI-3K in a GTP-dependent manner through the Ras effector site (Rodriguez-Viciana et al, 1994). Activation of PI-3K leads to activation of a serine/threonine kinase termed protein kinase B (PKB) or Akt (Downward, 1998), which promotes cell survival by phosphorylating and inactivating several key apoptosis regulatory molecules, including the pro-apoptotic bcl-2 family member Bad (Datta et al, 1997; del Peso et al, 1997), the protease caspase-9 (Cardone et al, 1998), and the forkhead transcription factor FKHRL1 (Brunet et al, 1999). In contrast to PI-3K, MAPK is traditionally considered to be a component of the Grb2/SOS/Ras/Raf/ERK protein kinase cascade, linking growth and differentiation signals with transcription in the nucleus. Activated MAPK (ERK p44/p42) translocates to the nucleus, where it activates transcription by phosphorylation of such transcription factors as Elk-1 and stat3. Recent studies suggested that MAPK is also involved in cell survival. Phosphorylation of Bad at either Ser-136 and Ser-112 promotes the binding of Bad to 14-3-3 protein and inhibits the binding of Bad to the pro-survival proteins Bcl-X and Bcl-2 (Zha et al, 1996). While Akt phosphorylates Bad at Ser-136, recent studies demonstrated that the MAPK-activated p90 ribosomal S6 kinase family (Rsks) phosphorylates Bad at Ser-112 (Bonni et al, 1999; Fang et al, 1999; Scheid et al, 1999). The results of these studies provided an important convergence of the Grb2/SOS/Ras/Raf/MEK/ MAPK/Rsk pathway and the PI-3K/Akt pathway in promoting cell survival.

We herein report our observations that EGF acts as a survival factor in inhibiting paclitaxel-induced apoptosis in SiHa human cervical cancer cells through a PI-3K- or MAPK-independent pathway. We found that the anti-apoptotic effect involves the EGF-activated c-Jun NH2-terminal kinase (JNK) pathway but not the PI-3K/Akt or MEK/MAPK signalling pathway in the cells. The JNK pathway is homologous to MAPK in its overall pathway but is activated largely by distinct extracellular stimuli, such as ultraviolet irradiation, osmotic stress, DNA-damaging agents, inflammatory cytokines and even growth factors (Ichijo, 1999; Leppa and Bohmann, 1999). EGF can activate the JNK signalling pathway in certain cell types (Hashimoto et al, 1999; Chen et al, 2000). In contrast to EGF-mediated MAPK activation, which was abolished upon the loss of the Grb2 adapter protein but not upon the loss of the Shc adaptor protein, EGF-mediated JNK activation

*Received 30 January 2001*
*Revised 30 April 2001*
*Accepted 3 May 2001*

*Correspondence to:* Z Fan was dependent on Shc but not on Grb2 (Hashimoto et al, 1999). Activation of JNK phosphorylates the N-terminal domain of the transcription factor c-Jun, thereby increasing its transactivation potency. Although there is compelling evidence that c-Jun activation can lead to apoptosis (Zanke et al, 1996; Tournier et al, 2000), a number of reports indicate that, under certain circumstances, activation of c-Jun can also inhibit apoptosis and promote cell proliferation, transformation, or differentiation (Nishina et al, 1997; Smith et al, 1997). In addition, many studies also report a lack of correlation between JNK activation and apoptosis (Liu et al, 1996; Khwaja and Downward, 1997). Thus, it is apparent that the effects of c-Jun activation on cellular response depend on cell types and the context of other regulatory signals that the cells receive from the environment. The results of our current studies indicate that activation of JNK by EGF protected SiHa cervical cancer cells from paclitaxel-induced apoptosis.

### MATERIALS AND METHODS

### Cell lines and tissue culture

SiHa human cervical cancer cells and MDA-MB-468 breast cancer cells were obtained from American Type Culture Collection (Manassas, VA, USA). The cells were maintained in 1:1 (v/v) Dulbecco's modified Eagle medium/Ham's F-12 mixture supplemented with 10% fetal bovine serum (FBS) in a 37˚C humidified atmosphere containing 95% air and 5% $CO_2$.

### Antibodies and reagents

Anti-HA monoclonal antibody was obtained from Roche Diagnostics Corp (Indianapolis, IN, USA). Anti-phosphotyrosine monoclonal antibody (4G10) was purchased from Upstate Biotechnology Inc (Lake Placid, NY, USA). Anti-phosphorylated Akt polyclonal antibodies (Ser473 and Thr308), anti-Akt polyclonal antibody, anti-phosphorylated p44/p42 MAPK monoclonal antibody, anti-phosphorylated JNK polyclonal antibody, anti-phosphorylated p38 MAPK polyclonal antibody, and anti-p38 MAPK polyclonal antibody were purchased from New England Biolabs, Inc (Beverly, MA, USA). Anti-ERK2 polyclonal antibody was from Santa Cruz Biotechnology, Inc (Santa Cruz, CA, USA), anti-JNK1 monoclonal antibody (G151–333) was from PharMingen Biotechnology, Inc (San Diego, CA, USA) and anti-poly-(ADP-ribose) polymerase (PARP) antibody C-2-10 was purchased from CHUL Research Center, Laval University (Quebec, Canada). Paclitaxel (Taxol) was purchased from Bristol-Myers Squibb Company (Princeton, NJ, USA). Recombinant EGF was obtained from Collaborative Research Inc. (Bedford, MA, USA). PD98059, LY294002, and SB202190 were obtained from CalBiochem Corp (San Diego, CA, USA). Protein A-sepharose beads used for immunoprecipitation were purchased from Repligen Corp (Cambridge, MA, USA). All other reagents were purchased from Sigma Chemical (St. Louis, MO, USA) unless otherwise specified.

### Quantification of apoptosis by ELISA

We used an apoptosis ELISA kit (Roche Diagnostics Corp) to quantitatively measure cytoplasmic histone-associated DNA fragments (mononucleosomes and oligonucleosomes) after induced cell death. This photomeric enzyme immunoassay was performed according to the manufacturer's instructions.

### Caspase-3 enzymatic activity assay

Caspase-3 enzymatic activities were measured by colorimetric assays with a kit purchased from Clontech Laboratories, Inc (Palo Alto, CA, USA). The assay is based on spectrophotometric detection of the chromophore *p*-nitroanilide (pNA), which is cleaved from the caspase-3 specific substrate DEVD-pNA by activated caspase-3. The assay was performed according to the manufacturer's instructions.

### Transfection of cells with expression vectors

Cell transfection was performed with the FuGENE™-6 transfection kit (Roche Diagnostic Corp) according to the manufacturer's instructions.

### Western blot analysis

Cells were lysed in a lysis buffer containing 50 mM Tris (pH 7.4), 150 mM NaCl, 0.5% NP-40, 50 mM NaF, 1 mM $Na_3VO_4$, 1 mM phenylmethylsulfonyl fluoride, 25 μg $ml^{-1}$ leupeptin, and 25 μg $ml^{-1}$ aprotinin. The lysates were cleared by centrifugation, and the supernatants were collected. Equal amounts of lysate protein were used for Western blot analysis with the indicated antibodies as previously described (Fan et al, 1995).

### PI-3K activity assay

The PI-3K activity assay was performed as previously reported (Lu et al, 1996). Briefly, equal amounts of cell lysate were subjected to immunoprecipitation with anti-phosphotyrosine monoclonal antibody 4G10. The immunoprecipitates were resuspended in 60 μl of kinase buffer containing 33 μM Tris-HCl (pH 7.6), 125 mM NaCl, 15 mM $MgCl_2$, 200 μM adenosine, 20 μM ATP, and 30 μCi of [$\gamma$-$^{32}$P]ATP (New England Nuclear, Boston, MA, USA). PI-3K assays were initiated by the addition of 10 μl of PI suspension to the immunoprecipitates. The reaction was allowed to proceed for 30 min at room temperature and was terminated by the addition of 100 μl of 1 N HCl to the reaction mixture. Lipids were extracted with 600 μl of chloroform-methanol (1:1) and separated by thin-layer chromotography with chloroform-methanol-ammonium hydroxide-distilled water (60:47:2:11.3). Radiolabelled PIP was visualized by autoradiography.

### JNK1 and p38 kinase activity assay

The JNK1 and p38 kinase assay was performed as previously described (Liu et al, 2000). Briefly, equal amounts of cell lysate were subjected to immunoprecipitation with anti-JNK1 monoclonal antibody or anti-p38 antibody. The immunoprecipitates were washed twice with a kinase buffer (20 mM Tris, 7.5 mM $MgCl_2$, 1 mM dithiothreitol). The kinase reaction was performed by incubating the immunoprecipitates with 40 μl of kinase buffer containing 2 μg of GST-c-Jun (or GST-ATF2), 25 μM lithium ATP, and 5 μCi of [$\gamma$-$^{32}$P]ATP at 30˚C for 30 min. The reaction was terminated by boiling the samples with 40 μl of 2x SDS sample buffer. The products of the reaction were resolved using 10% SDS-PAGE and then subjected to autoradiography.

 © 2001 Cancer Research Campaign

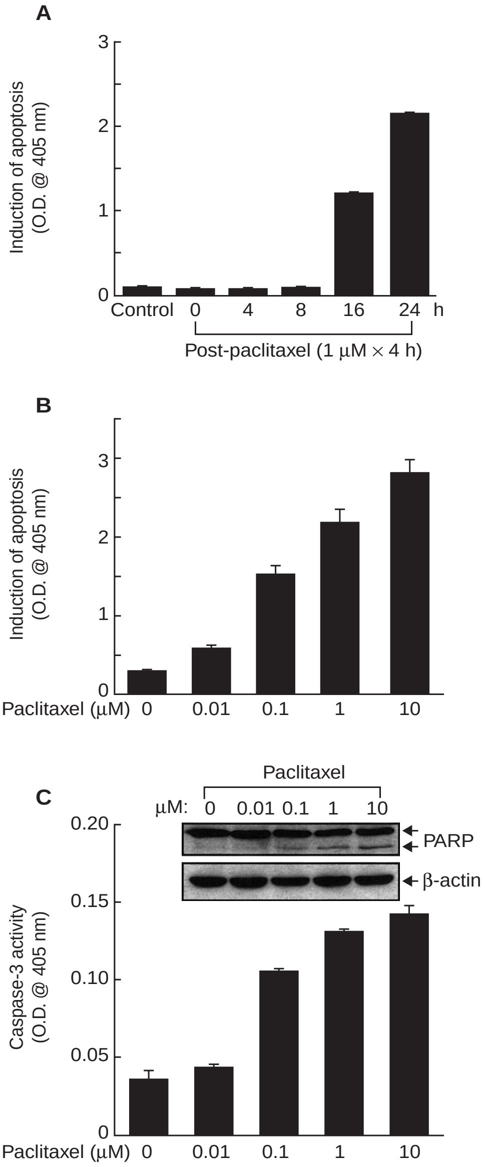

**Figure 1** Induction of apoptosis and activation of caspase-3 by paclitaxel in SiHa cells. SiHa cells were pulse-exposed to 1 μM paclitaxel for 4 h followed by additional indicated hours of post-paclitaxel period in culture medium containing 0.5% FBS (**A**), or were pulse-exposed to serially diluted doses of paclitaxel for 4 h, followed by a 20-h post-paclitaxel period in culture medium containing 0.5% FBS (**B** and **C**). Cells were then harvested and subjected to an apoptosis ELISA analysis (**A** and **B**) or caspase-3 assay (**C**) as described in Materials and Methods. Inset: Cleavage of the capsase-3 substrate PARP by paclitaxel treatment. Cell lysates were separated by SDS-PAGE, followed by Western blot analysis with antibodies against PARP and β-actin

### RESULTS

### Inhibition of paclitaxel-induced apoptosis by EGF in SiHa cells

SiHa cells are sensitive to treatment with paclitaxel. A 4-h pulse exposure of the cells to paclitaxel caused the cells to undergo apoptosis 16 to 24 h later, as measured by an apoptosis ELISA (Figure 1A). The induction of apoptosis was paclitaxel dose-dependent and was most pronounced in the dose range from 0.01 to 10 μM paclitaxel (Figure 1B). The apoptosis was characterized by an elevated level of caspase-3 activity and by cleavage of the caspase-3 substrate PARP (Figure 1C). When EGF was added during the post-paclitaxel period, the induction of apoptosis was markedly reduced (Figure 2A). The inhibition of paclitaxel-induced apoptosis by EGF was accompanied by a lower level of caspase-3 activity and a lower rate of cleavage of PARP (Figure 2B and inset).

### Lack of involvement of the PI-3K/Akt and MEK/MAPK pathways in EGF-mediated protection against paclitaxel-induced apoptosis

Because EGF activates the PI-3K/Akt and MEK/MAPK pathways in a variety of human cell types, to learn how EGF protects SiHa cells from paclitaxel-induced apoptosis, we first examined whether EGF activated one or both of these pathways. Figure 3 shows that SiHa cells contain a high basal level of phosphorylated MAPK and a high level of total ERK protein. In contrast with the results observed in a control cell line, MDA-MB-468 human breast cancer cells, that EGF stimulated phosphorylation of MAPK p44/p42, stimulation of SiHa cells with EGF under similar condition did not increase the MAPK phosphorylation. Figure 4A

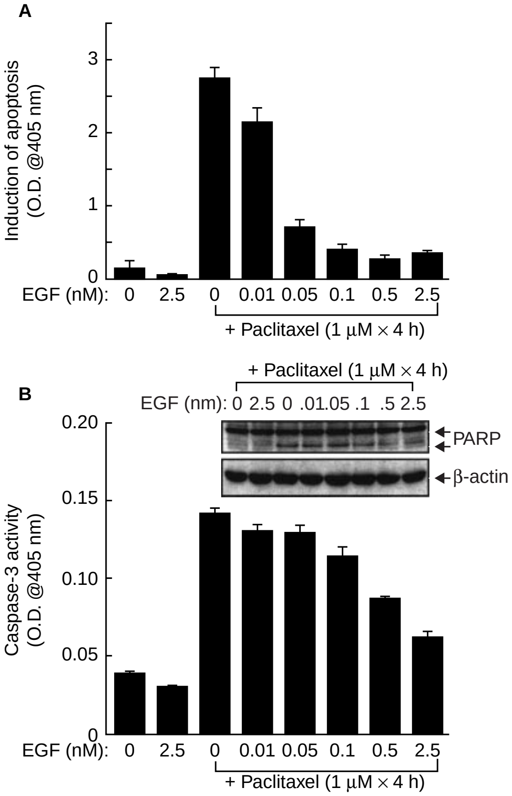

**Figure 2** Inhibition of paclitaxel-induced apoptosis and activation of caspase-3 by EGF in SiHa cells. SiHa cells were exposed to 1 μM paclitaxel for 4 h, followed by an additional 20-h post-paclitaxel culture period with increasing concentrations of EGF, in culture medium containing 0.5% FBS. Cells were then harvested and subjected to an apoptosis ELISA analysis (**A**) or caspase-3 assay (**B**) as described in Materials and Methods. Inset: Inhibition of paclitaxel-induced capsase-3 substrate PARP cleavage by EGF. Cell lysates were separated by SDS-PAGE, followed by Western blot analysis with antibodies against PARP and β-actin shows the results with a PI-3K activity assay. Again, in contrast to the results for MDA-MB-468 cells, in which EGF activated PI-3K (as shown by increased phosphorylation of phosphatidylinositols after EGF stimulation), SiHa cells exhibited a high basal level of PI-3K activity, and stimulation of these cells with EGF did not increase the level of phosphorylated phosphatidylinositols. In the MDA-MB-468 cells, Western blot analysis with anti-phosphorylated Akt antibodies (Ser473 or Thr308) showed a time-dependent phosphorylation of Akt upon EGF stimulation. Despite the high basal level of PI-3K activity, the level of phosphorylated Akt was minimal in SiHa cells, even though the cells did display a significant level of total Akt protein shown as a doublet band on the SDS-electrophoresis pattern (Figure 4B). Western blot analysis with an Akt2 specific antibody indicated that the lower band of the Akt doublet was in same position as Akt2, suggesting that it is likely to represent Akt2 (data not shown). The observation that stimulation of SiHa cells with EGF did not produce any detectable change in phosphorylated Akt on serine-473 or threonine-308 suggests that there might be a defect in the signal transduction pathway leading to phosphorylation of Akt upon PI-3K activation in SiHa cells, which is beyond the scope of current study. Taken together, these results indicate that EGF does not stimulate the PI-3K/Akt and MEK/MAPK pathways in SiHa cells.

The PI-3K-specific inhibitor LY294002 and the MEK-specific inhibitor PD98059 have been extensively used in literature for their respective specific effects on these two pathways (Vlahos et al, 1994, 1995; Dudley et al, 1995; Langlois et al, 1995; Waters et al, 1995; Yano et al, 1995; Baumann and West, 1998; Cardone et al, 1998; Kultz et al, 1998). To exclude whether any basal activities of PI-3K/Akt and MEK/MAPK were involved in EGF-mediated protection against paclitaxel-induced apoptosis, we investigated whether these two inhibitors could interfere with this protection. Figure 5A shows that the paclitaxel-induced apoptosis was strongly inhibited by EGF (Figure 5A, bars 5 and 6). Co-exposure of the cells to EGF and LY294002 (bar 7) or PD98059 (bar 8) had only a moderate effect on EGF-mediated inhibition of paclitaxel-induced apoptosis. To further confirm this result, we examined the effects of transient expression of dominant-negative MEK cDNA (MEK-DN) or dominant-negative PI-3K (Δp85) on the EGF-mediated inhibition of paclitaxel-induced apoptosis. Both MEK-DN and PI-3K Δp85 were expressed in SiHa cells after the transient transfection (Figure 5B, inset). In our studies, transfection of the cells with an expression vector containing GFP cDNA under similar experimental conditions resulted in 35–50% of the cells being GFP positive (green cells) (data not shown). Neither the expression of MEK-DN nor the expression of PI-3K Δp85 reversed the protective effect of EGF against paclitaxel-induced apoptosis in the SiHa cells (Figure 5B).

### Involvement of JNK activity in the EGF-mediated protection against paclitaxel-induced apoptosis

JNK plays a dual role in the regulation of apoptosis. Recent studies have shown that JNK can be involved in both the induction and suppression of apoptosis in response to a variety of death signals (Leppa and Bohmann, 1999). Because EGF can activate JNK under some circumstances (Hashimoto et al, 1999; Chen et al, 2000), we therefore examined whether EGF activated JNK and the related p38 MAPK in SiHa cells. We found that exposure of SiHa cells to EGF resulted in a transient enhanced phosphorylation of both JNK1 and JNK2, which peaked around 10 min after EGF stimulation and disappeared 1 to 2 h after EGF stimulation (Figure 6A). There was no change in the level of JNK1 protein upon EGF stimulation. We further confirmed this result with an in vitro kinase assay using GST-Jun fusion protein as a substrate to measure immunoprecipitated JNK1 activity following EGF treatment in SiHa cells. In contrast to the results obtained with JNK activation, EGF only marginally affected p38 MAPK phosphorylation and did not change its expression level in the cells (Figure 6B).

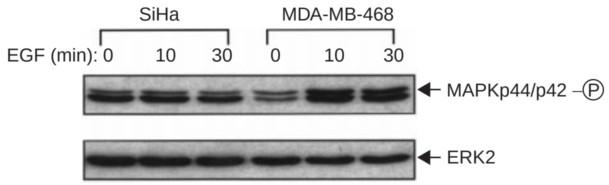

**Figure 3** Effect of EGF on activation of MEK/MAPK in SiHa cells. SiHa and MDA-MB-468 cells were untreated or treated with 5 nM EGF for 10 and 30 min. Cells were then harvested, lysed, and subjected to Western blot analyses with antibodies against phosphorylated MAPK p44/p42 and total ERK2

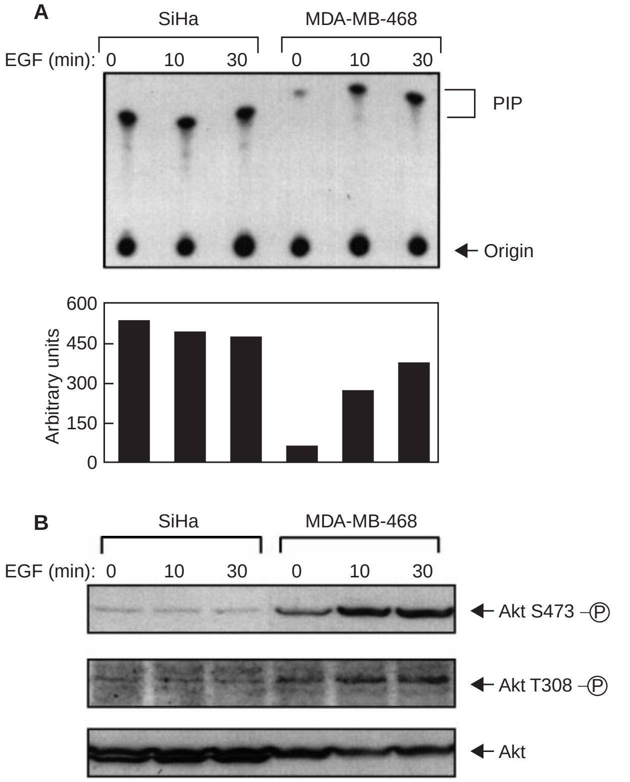

**Figure 4** Effect of EGF on activation of PI-3K/Akt in SiHa cells. **A**, SiHa and MDA-MB-468 cells were untreated or treated with 5 nM EGF for 10 and 30 min. Cells were then harvested, lysed, and subjected to immunoprecipitation with the anti-phosphotyrosine monoclonal antibody 4G10. The immunoprecipitates were assayed for PI-3K activity with phosphatidylinositol (PI) as a substrate as described in Materials and Methods. The bar graph underneath shows quantitative determination (using arbitrary units) of PIP production by phosphoimaging analysis. **B**, SiHa and MDA-MB-468 cells were treated as described in (**A**). Cell lysates were prepared and subjected to Western blot analysis with antibodies against ser473-phosphorylated Akt1, thr308-phosphorylated Akt1, and total Akt protein, respectively

 © 2001 Cancer Research Campaign

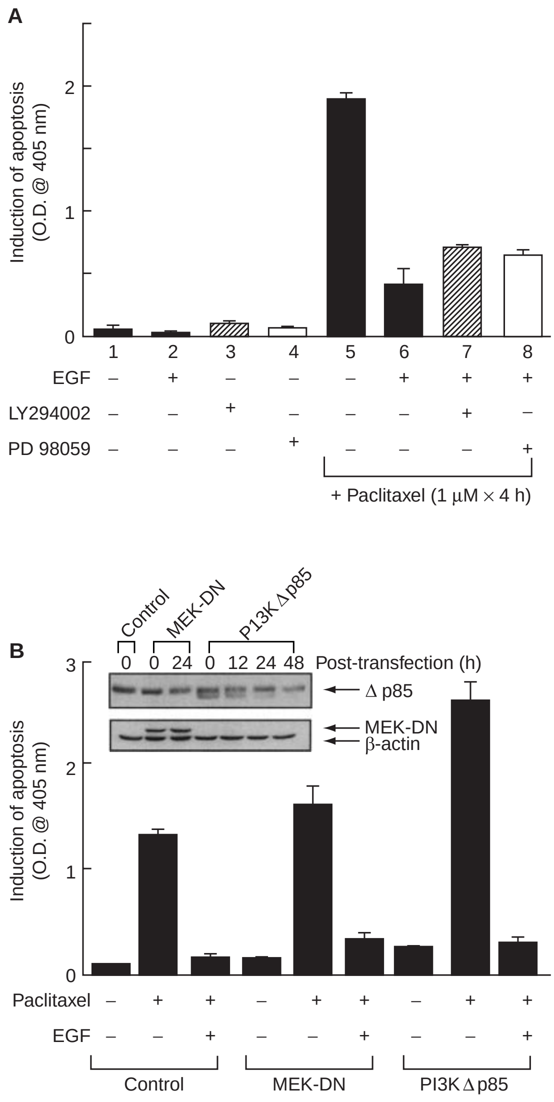

**Figure 5** Lack of PI-3K/Akt and MEK/MAPK signalling pathway involvement in EGF-mediated protection from paclitaxel-induced apoptosis in SiHa cells. **A**, In the left 4 bars, SiHa cells were cultured for 20 h with no addition (control), 5 nM EGF, 2 μM LY 294002, or 10 μM PD 98059; in the right 4 bars, SiHa cells were pulse-exposed to 1 μM paclitaxel for 4 h, followed by a 20-h post-paclitaxel period of culture with no addition, 5 nM EGF, 5 nM EGF plus 2 μM LY 294002, or 5 nM EGF plus 10 μM PD 98059, as indicated. Cells were harvested and subjected to an apoptosis ELISA analysis. **B**, SiHa cells were transiently transfected for 30 h with a control vector, an HA-tagged MEK dominant-negative (MEK-DN) vector, or an HA-tagged PI-3K dominant-negative (Δ p85) vector. During the last 4 h of the transfection, cells were pulse-exposed to 1 μM paclitaxel. The cells were then cultured for an additional 20-h post-paclitaxel period in the absence or presence of 5 nM EGF, in 0.5% FBS medium. Cells were harvested and subjected to an apoptosis ELISA analysis. Inset: Expression of PI-3K dominant-negative vector (Δ p85) and MEK dominant-negative vector (MEK-DN). After the plasmids were removed from the culture medium, the cells were either harvested immediately (0 h) or cultured for additional hours in regular culture medium. Equal amounts of lysate protein from each sample were subjected to Western blot analysis for the expression of MEK-DN or Δ p85 with anti-HA or β-actin antibody

An in vitro kinase assay using GST-ATF2 fusion protein as a substrate showed no change in the phosphorylation level of GST-ATF2 following EGF treatment in SiHa cells.

To determine whether the JNK activation contributed to the EGF-mediated protection against paclitaxel-induced apoptosis, we examined whether selective inhibition of the JNK pathway with

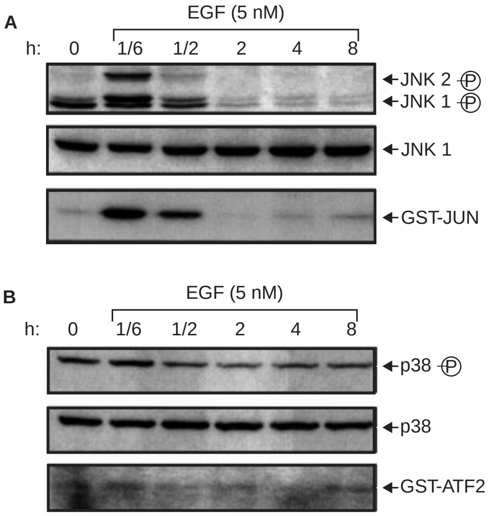

**Figure 6** Activation of JNK but not p38 MAPK by EGF in SiHa cells. **A**, SiHa cells were untreated or treated with 5 nM EGF for the indicated time intervals. Cells were then harvested, lysed, and subjected to Western blot analysis with antibodies that recognize phosphorylated isoforms of all 3 JNKs, and antibodies that specifically recognize JNK1, and then subjected to an in vitro JNK1 kinase assay using GST-Jun as a substrate, as described in Materials and Methods. **B**, SiHa cells were untreated or treated with 5 nM EGF for the indicated time intervals. Cells were then harvested, lysed, and subjected to Western blot analysis with antibodies against phosphorylated p38 MAPK and total p38 MAPK and then subjected to an in vitro p38 kinase assay using GST-ATF2 as a substrate, as described in Materials and Methods the pyridinyl imidazole compound SB202190 would reverse the protection. SB202190 was initially identified as a specific inhibitor for p38 MAPK (Lee et al, 1994) but recent studies have indicated that SB202190 also blocks activation of the JNK pathway (Chen et al, 1998; Ming et al, 1998). Thus, we first examined whether SB202190 could block JNK activation induced by ultraviolet irradiation in SiHa cells. We found that SB202190 clearly inhibited the activities of JNK1 and JNK2 in an SB202190 dose-dependent manner (Figure 7A). There was no change in the level of JNK1 protein upon ultraviolet irradiation and SB202190 treatment. An in vitro kinase assay with GST-Jun fusion protein as a substrate showed similar result of JNK activity inhibition by SB202190. We then examined the effect of SB202190 on EGF-induced activation of JNK and EGF-mediated protection against paclitaxel-induced apoptosis in the cells. Pretreatment of SiHa cells with 20 μM SB202190 inhibited EGF-induced JNK1 activation at both the 10-min and 30-min time points (Figure 7B). Pre-exposure of SiHa cells to 20 μM SB202190 almost completely reversed the protective effect of EGF (Figure 7C). The reversal of EGF-mediated protection against paclitaxel-induced apoptosis by SB202190 was accompanied by restoration of caspase-3 activity and cleavage of the caspase-3 substrate PARP (Figure 7D and 7E). Because EGF did not activate p38 MAPK in SiHa cells (Figure 6B), our results therefore strongly suggest that JNK activation is involved in the EGF-mediated inhibition of paclitaxel-induced apoptosis in SiHa cells.

### DISCUSSION

In this article, we report our results elucidating the signal pathways by which EGF protects SiHa cervical carcinoma cells from paclitaxel-induced apoptosis. In contrast to its well-documented

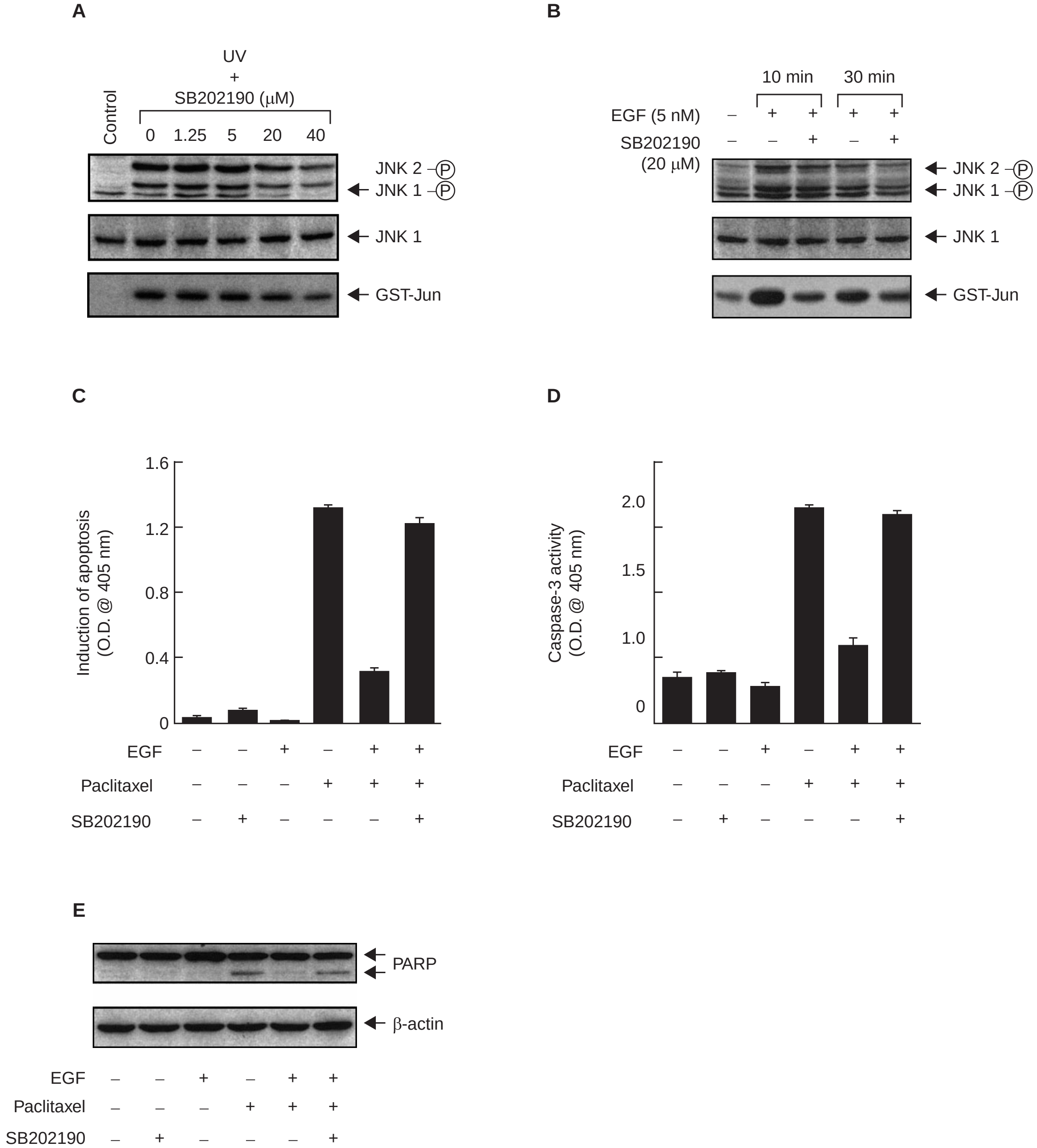

**Figure 7** Abrogation of EGF-mediated protection from paclitaxel-induced apoptosis in SiHa cells by SB202190. **A**, Dose-dependent inhibition of ultraviolet irradiation-induced JNK phosphorylation and activation by SB202190. Following ultraviolet irradiation, SiHa cells were cultured for 1 h in the presence or absence of the indicated doses of SB202190. Cells were then harvested, lysed, and then subjected to Western blot analysis with antibodies against phosphorylated JNK and total JNK1 and subjected to an in vitro JNK1 kinase assay using GST-Jun as a substrate, as described in Materials and Methods. **B**, Inhibition of EGF-activated JNK1 by SB202190. Cells were pretreated with 20 μM SB202190 for 1 h, followed by stimulation of the cells with 5 nM EGF for 10 and 30 min. Cell lysates were prepared and analysed as described in (**A**). **C**, **D** and **E**, Reversal of EGF-mediated inhibition of apoptosis by SB202190. SiHa cells were exposed to 1 μM paclitaxel for 4 h, followed by an additional 20-h post-paclitaxel culture period in the absence or presence of 5 nM EGF or of 5 nM EGF plus 20 μM SB202190, as indicated. Cell lysates were prepared and subjected to an apoptosis ELISA analysis (**C**), caspase-3 activity assay (**D**), or Western blotting analysis with antibodies directed against PARP and β-actin (**E**)

activation of PI-3K/Akt or MEK/MAPK pathways in other cells, EGF inhibited apoptosis in SiHa cells through a mechanism that involves JNK activity. Overall, whether or not the JNK pathway operates as a major EGF-mediated protective pathway in human cancer seems cell type-dependent which is apparently the case in SiHa cells. We speculate that it may exist as a backup pathway in parallel with the MAPK and Akt pathways in some types of cells or under certain circumstances.

SiHa cells have low EGF receptor density compared with other squamous carcinoma cell lines such as A431, HN5 or Caski. We found that SiHa cells appear defective in Akt phosphorylation following stimulation of the cells with EGF. The reasons why EGF failed to stimulate PI-3K in SiHa cells and why the high basal level of PI-3K was accompanied only by minimal level of Akt phosphorylation were not explored in current study, because this would deviate from our focus. The lack of effect of EGF stimulation on

 © 2001 Cancer Research Campaign

PI-3K/Akt could be due to low expression of HER3 in these cells (data not shown), which is generally believed to be a necessary intermediate to couple the EGF receptor to this pathway. The failure of Akt phosphorylation in the cells could be due to a possibility that the kinases that phosphorylate Akt at threonine 308 and serine 473 (PDK1 and PDK2, respectively) are defective. Alternatively SiHa cells might express mutated Akt proteins that can not be phosphorylated by PDK1 and PDK2. In addition to having an abnormality in the PI-3K/Akt pathway, the SiHa cells did not show a typical response to EGF-mediated activation of MAPK either, although the proliferation of SiHa cells was stimulated by EGF. Our as-yet-unpublished results indicate that EGF appears to stimulate SiHa cell proliferation through a mechanism that is independent of cyclin-dependent kinase activity (Schmidt and Fan, manuscript in review). Inducible expression of $p16^{Ink4a}$, $p21^{Waf1}$ or $p27^{Kip1}$ in these cells, although strongly inhibiting CDK activity, could not override the stimulatory effect of EGF on cell proliferation, presumably because of the HPV16 infection status in these cells. The HPV viral oncoprotein E7 has been shown to render cells capable to bypass G1 arrest induced by serum deprivation and by $p21^{Waf1}$, because the E7 protein constitutively inactivates the Rb protein and causes sequestration of Rb from E2F binding (Morozov et al, 1997).

After we determined that the PI-3K/Akt and MEK/MAPK pathways were not involved, we examined the possible involvement of the JNK pathway and found that the mechanism by which EGF protected SiHa cells from paclitaxel-induced apoptosis was sensitive to inhibition of JNK activity by SB202190. Although EGF did not increase the activity of p38 MAPK (Figure 6B), our results shown in Figure 6B do not exclude a possible requirement of some basal activity of p38 MAPK for EGF-mediated protection against paclitaxel-induced apoptosis, because the dose of SB202190 used to inhibit JNK1 activity (Figure 6A) can also inhibit the basal activity of p38 MAPK (Ming et al, 1998).

The PI-3K pathway has been implicated in the activation of the JNK signalling pathway (Klippel et al, 1996; Logan et al, 1997). In these previous studies, EGF activated JNK1 in the HPV18-positive HeLa human cervical cancer cell line, and this activation was blocked by treatment of the cells with the PI-3K inhibitor wortmannin and by transfection of the cells with a PI-3K dominant-negative expression vector, suggesting that PI-3K played a role in EGF-induced JNK activation in HeLa cells. Similarly, overexpression of a truncated EGF receptor, EGFRvIII, transformed NIH3T3 cells, accompanied by constitutive activation of PI-3K and JNK1, with no increase in Ras/GTP levels and with low levels of MAPK activity (Huang et al, 1997; Antonyak et al, 1998; Moscatello et al, 1998). This constitutive JNK activity was downregulated following treatment of the cells with the PI-3K specific inhibitor LY294002 (Treisman, 1996). The results of our current study, however, suggest that EGF-induced JNK activation is PI-3K-independent. Previous studies have also shown that, in addition to PI-3K, Ras and the Ras-related Rac/Rho small GTP-binding proteins can also mediate EGF-induced JNK activation (Su and Karin, 1996). EGF-mediated JNK activation was inhibited by dominant negative Ras (RasN17) and dominant negative Rac1 (Rac1N17) (Wood et al, 1992; Susin et al, 1999). There are at least two possible signalling pathways by which the EGF receptor can activate Ras: one is the direct binding of the Grb2/SOS/Ras complex to the phosphorylated EGF receptor (Li et al, 1993; Batzer et al, 1994), and the other pathway involves the Shc adaptor protein (Shc/Grb2/SOS/Ras) (Rozakis-Adcock et al, 1992; Gotoh et al, 1995). Our observation that EGF activated JNK activity in the SiHa cells without affecting the activities of PI-3K, ERK and p38 MAPK suggests that JNK activation by EGF in SiHa cells might involve, although not necessarily, the Shc adaptor protein.

The mechanism by which JNK-mediated pathway inhibits paclitaxel-induced apoptosis in SiHa cells may partially involve enhanced degradation of p53, because inhibition of EGF-induced JNK activation with the JNK inhibitor SB202190 was accompanied by reduced degradation of p53 and reduced inhibition of the paclitaxel-induced apoptosis by EGF (data not shown). In addition, inhibition of p53 degradation in SiHa cells with the 26S proteasaome inhibitor MG132 could partially reverse paclitaxel-induced apoptosis (data not shown). It is known that EGF can activate AP-1, which is a collection of dimeric sequence-specific transcriptional factors composed of c-Jun and c-Fos, in SiHa cells and that AP-1 can bind to the enhancer region of HPV E6/E7 genes, thereby increasing the levels of HPV E6 and E7 expression (Peto et al, 1995). Increased expression of E6 would then result in increased binding to the E6-associated protein (E6-AP), and the complex would tightly associate with p53, leading to rapid degradation of p53 via a ubiquitin proteasome-dependent pathway (Scheffner et al, 1990; Crook et al, 1991). Unfortunately, we were not able to detect E6 protein with Western blot analysis in our study, presumably because of the very low concentrations of E6 protein produced by the naturally infected virus in SiHa cells. Previous studies used Northern blot analysis to measure changes in the HPV E6/E7 mRNA level in HPV-infected cells. HPV E6/E7 protein was detected by Western blot analysis only in HPV E6/E7 cDNA-transfected cells.

As it was mentioned in the introduction, JNK1 appears to play a critical role in paclitaxel-induced apoptosis in several cellular systems. Paclitaxel activates ASK1/JNK1, Raf/MAPK and p38 MAPK that may contribute to Bcl2 phosphorylation and release of Bax resulting in apoptosis in these cellular systems (Stone and Chambers, 2000; Subbaramaiah et al, 2000). These results appear to contradict our results in the current study; however, there are clearly JNK-independent mechanisms by which paclitaxel induces apoptosis (Wang et al, 1999). SiHa cervical carcinoma cells, due to the presence of HPV E6 and EGF-induced enhancement of E6 expression and subsequent degradation of p53, may represent a different paradigm, wherein, JNK1-dependent p53 degradation through JNK1/AP-1/E6/p53 plays a dominant role in determining whether the cells undergo apoptosis. The result suggests that JNK1 may play different roles in paclitaxel-induced apoptosis in different cell lineages.

In summary, we demonstrated that the JNK signalling pathway plays an important role in EGF-mediated protection from paclitaxel-induced apoptosis in the HPV E6-expressing SiHa cells. Our data suggest that there could be clinical benefits from appropriate combination of conventional chemotherapeutic drugs with new generation of signal transduction inhibitors.

### ACKNOWLEDGEMENTS

The authors are grateful to Mr Michael Worley of the Department of Scientific Publications for editorial assistance with the manuscript. This work was supported in part by a start-up fund to ZF from The University of Texas MD Anderson Cancer Center, a research award from Bristol-Myers Squib Company and by the NCI Cancer Center Core Grant CA16672.

© 2001 Cancer Research Campaign 

**REFERENCES**

Antonyak MA, Moscatello DK and Wong AJ (1998) Constitutive activation of c-Jun N-terminal kinase by a mutant epidermal growth factor receptor. *J Biol Chem* **273**: 2817–2822

Batzer AG, Rotin D, Urena JM, Skolnik EY and Schlessinger J (1994) Hierarchy of binding sites for Grb2 and Shc on the epidermal growth factor receptor. *Mol Cell Biol* **14**: 5192–5201

Baumann P and West SC (1998) DNA end-joining catalyzed by human cell-free extracts. *Proc Natl Acad Sci USA* **95**: 14066–14070

Bonni A, Brunet A, West AE, Datta SR, Takasu MA and Greenberg ME (1999) Cell survival promoted by the Ras-MAPK signaling pathway by transcription-dependent and -independent mechanisms. *Science* **286**: 1358–1362

Brunet A, Bonni A, Zigmond MJ, Lin MZ, Juo P, Hu LS, Anderson MJ, Arden KC, Blenis J and Greenberg ME (1999) Akt promotes cell survival by phosphorylating and inhibiting a Forkhead transcription factor. *Cell* **96**: 857–868

Caraglia M, Abbruzzese A, Leardi A, Pepe S, Budillon A, Baldassare G, Selleri C, Lorenzo SD, Fabbrocini A, Giuberti G, Vitale G, Lupoli G, Bianco AR and Tagliaferri P (1999) Interferon-alpha induces apoptosis in human KB cells through a stress-dependent mitogen activated protein kinase pathway that is antagonized by epidermal growth factor. *Cell Death Differ* **6**: 773–780

Cardone MH, Roy N, Stennicke HR, Salvesen GS, Franke TF, Stanbridge E, Frisch S and Reed JC (1998) Regulation of cell death protease caspase-9 by phosphorylation. *Science* **282**: 1318–1321

Chen BK, Kung HC, Tsai TY and Chang WC (2000) Essential role of mitogen-activated protein kinase pathway and c-Jun induction in epidermal growth factor-induced gene expression of human 12-lipoxygenase. *Mol Pharmacol* **57**: 153–161

Chen CY, Gatto-Konczak F, Wu Z and Karin M (1998) Stabilization of interleukin-2 mRNA by the c-Jun NH2-terminal kinase pathway. *Science* **280**: 1945–1949

Crook T, Tidy JA and Vousden KH (1991) Degradation of p53 can be targeted by HPV E6 sequences distinct from those required for p53 binding and trans-activation. *Cell* **67**: 547–556

Datta SR, Dudek H, Tao X, Masters S, Fu H, Gotoh Y and Greenberg ME (1997) Akt phosphorylation of BAD couples survival signals to the cell-intrinsic death machinery. *Cell* **91**: 231–241

del Peso L, Gonzalez-Garcia M, Page C, Herrera R and Nunez G (1997) Interleukin-3-induced phosphorylation of BAD through the protein kinase Akt. *Science* **278**: 687–689

Downward J (1998) Mechanisms and consequences of activation of protein kinase B/Akt. *Curr Opin Cell Biol* **10**: 262–267

Dudley DT, Pang L, Decker SJ, Bridges AJ and Saltiel AR (1995) A synthetic inhibitor of the mitogen-activated protein kinase cascade. *Proc Natl Acad Sci USA* **92**: 7686–7689

Fan Z, Lu Y, Wu X, DeBlasio A, Koff A and Mendelsohn J (1995) Prolonged induction of $p21^{Cip1/WAF1}$/CDK2/PCNA complex by epidermal growth factor receptor activation mediates ligand-induced A431 cell growth inhibition. *J Cell Biol* **131**: 235–242

Fang X, Yu S, Eder A, Mao M, Bast RC, Jr., Boyd D and Mills GB (1999) Regulation of BAD phosphorylation at serine 112 by the Ras-mitogen-activated protein kinase pathway. *Oncogene* **18**: 6635–6640

Gibson S, Tu S, Oyer R, Anderson SM and Johnson GL (1999) Epidermal growth factor protects epithelial cells against Fas-induced apoptosis. Requirement for Akt activation. *J Biol Chem* **274**: 17612–17618

Gotoh N, Muroya K, Hattori S, Nakamura S, Chida K and Shibuya M (1995) The SH2 domain of Shc suppresses EGF-induced mitogenesis in a dominant negative manner. *Oncogene* **11**: 2525–2533

Hashimoto A, Kurosaki M, Gotoh N, Shibuya M and Kurosaki T (1999) Shc regulates epidermal growth factor-induced activation of the JNK signaling pathway. *J Biol Chem* **274**: 20139–20143

Huang HS, Nagane M, Klingbeil CK, Lin H, Nishikawa R, Ji XD, Huang CM, Gill GN, Wiley HS and Cavenee WK (1997) The enhanced tumorigenic activity of a mutant epidermal growth factor receptor common in human cancers is mediated by threshold levels of constitutive tyrosine phosphorylation and unattenuated signaling. *J Biol Chem* **272**: 2927–2935

Ichijo H (1999) From receptors to stress-activated MAP kinases. *Oncogene* **18**: 6087–6093

Khwaja A and Downward J (1997) Lack of correlation between activation of Jun-NH2-terminal kinase and induction of apoptosis after detachment of epithelial cells. *J Cell Biol* **139**: 1017–1023

Klippel A, Reinhard C, Kavanaugh WM, Apell G, Escobedo MA and Williams LT (1996) Membrane localization of phosphatidylinositol 3-kinase is sufficient to activate multiple signal-transducing kinase pathways. *Mol Cell Biol* **16**: 4117–4127

Kultz D, Madhany S and Burg MB (1998) Hyperosmolality causes growth arrest of murine kidney cells. Induction of GADD45 and GADD153 by osmosensing via stress-activated protein kinase 2. *J Biol Chem* **273**: 13645–13651

Lan L and Wong NS (1999) Phosphatidylinositol 3-kinase and protein kinase C are required for the inhibition of caspase activity by epidermal growth factor. *FEBS Lett* **444**: 90–96

Langlois WJ, Sasaoka T, Saltiel AR and Olefsky JM (1995) Negative feedback regulation and desensitization of ins. *J Biol Chem* **270**: 25320–25323

Lee JC, Laydon JT, McDonnell PC, Gallagher TF, Kumar S, Green D, McNulty D, Blumenthal MJ, Heys JR and Landvatter SW (1994) A protein kinase involved in the regulation of inflammatory cytokine biosynthesis. *Nature* **372**: 739–746

Leppa S and Bohmann D (1999) Diverse functions of JNK signaling and c-Jun in stress response and apoptosis. *Oncogene* **18**: 6158–6162

Leu CM, Chang C and Hu C (2000) Epidermal growth factor (EGF) suppresses staurosporine-induced apoptosis by inducing mcl-1 via the mitogen-activated protein kinase pathway. *Oncogene* **19**: 1665–1675

Li N, Batzer A, Daly R, Yajnik V, Skolnik E, Chardin P, Bar-Sagi D, Margolis B and Schlessinger J (1993) Guanine-nucleotide-releasing factor hSos1 binds to Grb2 and links receptor tyrosine kinases to Ras signalling. *Nature* **363**: 85–88

Liu B, Fang M, Schmidt M, Lu Y, Mendelsohn J and Fan Z (2000) Induction of apoptosis and activation of the caspase cascade by anti-EGF receptor monoclonal antibodies in DiFi human colon cancer cells do not involve the c-jun N-terminal kinase activity. *Br J Cancer* **82**: 1991–1999

Liu ZG, Hsu H, Goeddel DV and Karin M (1996) Dissection of TNF receptor 1 effector functions: JNK activation is not linked to apoptosis while NF-kappaB activation prevents cell death. *Cell* **87**: 565–576

Logan SK, Falasca M, Hu P and Schlessinger J (1997) Phosphatidylinositol 3-kinase mediates epidermal growth factor-induced activation of the c-Jun N-terminal kinase signaling pathway. *Mol Cell Biol* **17**: 5784–5790

Lu Y, Rodriguez R, Bjorndahl J, Phillips CA and Trevillyan JM (1996) CD28-dependent killing by human YT cells requires phosphatidylinositol 3-kinase activation. *Eur J Immunol* **26**: 1278–1284

McClellan M, Kievit P, Auersperg N and Rodland K (1999) Regulation of proliferation and apoptosis by epidermal growth factor and protein kinase C in human ovarian surface epithelial cells. *Exp Cell Res* **246**: 471–479

Ming XF, Kaiser M and Moroni C (1998) c-jun N-terminal kinase is involved in AUUUA-mediated interleukin-3 mRNA turnover in mast cells. *EMBO J* **17**: 6039–6048

Morozov A, Shiyanov P, Barr E, Leiden JM and Raychaudhuri P (1997) Accumulation of human papillomavirus type 16 E7 protein bypasses G1 arrest induced by serum deprivation and by the cell cycle inhibitor p21. *J Virol* **71**: 3451–3457

Moscatello DK, Holgado-Madruga M, Emlet DR, Montgomery RB and Wong AJ (1998) Constitutive activation of phosphatidylinositol 3-kinase by a naturally occurring mutant epidermal growth factor receptor. *J Biol Chem* **273**: 200–206

Nishina H, Fischer KD, Radvanyi L, Shahinian A, Hakem R, Rubie EA, Bernstein A, Mak TW, Woodgett JR and Penninger JM (1997) Stress-signalling kinase Sek1 protects thymocytes from apoptosis mediated by CD95 and CD3. *Nature* **385**: 350–353

Payne SG, Brindley DN and Guilbert LJ (1999) Epidermal growth factor inhibits ceramide-induced apoptosis and lowers ceramide levels in primary placental trophoblasts. *J Cell Physiol* **180**: 263–270

Peto M, Tolle-Ersu I, Kreysch HG and Klock G (1995) Epidermal growth factor induction of human papillomavirus type 16 E6/E7 MRNA in tumor cells involves two AP-1 binding sites in the viral enhancer. *J Gen Virol* **76** (Pt 8): 1945–1958

Rodrigues GA, Falasca M, Zhang Z, Ong SH and Schlessinger J (2000) A novel positive feedback loop mediated by the docking protein Gab1 and phosphatidylinositol 3-kinase in epidermal growth factor receptor signaling. *Mol Cell Biol* **20**: 1448–1459

Rodriguez-Viciana P, Warne PH, Dhand R, Vanhaesebroeck B, Gout I, Fry MJ, Waterfield MD and Downward J (1994) Phosphatidylinositol-3-OH kinase as a direct target of Ras. *Nature* **370**: 527–532

Rozakis-Adcock M, McGlade J, Mbamalu G, Pelicci G, Daly R, Li W, Batzer A, Thomas S, Brugge J and Pelicci PG (1992) Association of the Shc and Grb2/Sem5 SH2-containing proteins is implicated in activation of the Ras pathway by tyrosine kinases. *Nature* **360**: 689–692

Scheffner M, Werness BA, Huibregtse JM, Levine AJ and Howley PM (1990) The E6 oncoprotein encoded by human papillomavirus types 16 and 18 promotes the degradation of p53. *Cell* **63**: 1129–1136

Scheid MP, Schubert KM and Duronio V (1999) Regulation of bad phosphorylation and association with Bcl-x(L) by the MAPK/Erk kinase. *J Biol Chem* **274**: 31108–31113

 *© 2001 Cancer Research Campaign*

Smith A, Ramos-Morales F, Ashworth A and Collins M (1997) A role for JNK/SAPK in proliferation, but not apoptosis, of IL-3-dependent cells. *Curr Biol* **7**: 893–896

Stone AA and Chambers TC (2000) Microtubule inhibitors elicit differential effects on MAP kinase (JNK, ERK, and p38) signaling pathways in human KB-3 carcinoma cells. *Exp Cell Res* **254**: 110–119

Su B and Karin M (1996) Mitogen-activated protein kinase cascades and regulation of gene expression. *Curr Opin Immunol* **8**: 402–411

Subbaramaiah K, Hart JC, Norton L and Dannenberg AJ (2000) Microtubule-interfering agents stimulate the transcription of cyclooxygenase-2. Evidence for involvement of ERK1/2 and p38 mitogen-activated protein kinase pathways. *J Biol Chem* **275**: 14838–14845

Susin SA, Lorenzo HK, Zamzami N, Marzo I, Snow BE, Brothers GM, Mangion J, Jacotot E, Costantini P, Loeffler M, Larochette N, Goodlett DR, Aebersold R, Siderovski DP, Penninger JM and Kroemer G (1999) Molecular characterization of mitochondrial apoptosis-inducing factor. *Nature* **397**: 441–446

Tournier C, Hess P, Yang DD, Xu J, Turner TK, Nimnual A, Bar-Sagi D, Jones SN, Flavell RA and Davis RJ (2000) Requirement of JNK for stress-induced activation of the cytochrome c-mediated death pathway. *Science* **288**: 870–874

Treisman R (1996) Regulation of transcription by MAP kinase cascades. *Curr Opin Cell Biol* **8**: 205–215

Ullrich A and Schlessinger J (1990) Signal transduction by receptors with tyrosine kinase activity. *Cell* **61**: 203–212

Vlahos CJ, Matter WF, Hui KY and Brown RF (1994) A specific inhibitor of phosphatidylinositol 3-kinase, 2-(4-morpholinyl)-8-phenyl-4H-1-benzopyran-4-one (LY294002). *J Biol Chem* **269**: 5241–5248

Vlahos CJ, Matter WF, Brown RF, Traynor-Kaplan AE, Heyworth PG, Prossnitz ER, Ye RD, Marder P, Schelm JA and Rothfuss KJ (1995) Investigation of neutrophil signal transduction using a specific inhibitor of phosphatidylinositol 3-kinase. *J Immunol* **154**: 2413–2422

Wang TH, Popp DM, Wang HS, Saitoh M, Mural JG, Henley DC, Ichijo H and Wimalasena J (1999) Microtubule dysfunction induced by paclitaxel initiates apoptosis through both c-Jun N-terminal kinase (JNK)-dependent and -independent pathways in ovarian cancer cells. *J Biol Chem* **274**: 8208–8216

Waters SB, Holt KH, Ross SE, Syu LJ, Guan KL, Saltiel AR, Koretzky GA and Pessin JE (1995) Desensitization of Ras activation by a feedback disassociation of the SOS-Grb2 complex. *J Biol Chem* **270**: 20883–20886

Wood KW, Sarnecki C, Roberts TM and Blenis J (1992) Ras mediates nerve growth factor receptor modulation of three signal-transducing protein kinases: MAP kinase, Raf-1, and RSK. *Cell* **68**: 1041–1050

Yano H, Agatsuma T, Nakanishi S, Saitoh Y, Fukui Y, Nonomura Y and Matsuda Y (1995) Biochemical and pharmacological studies with KT7692 and LY294002 on the role of phosphatidylinositol 3-kinase in Fc epsilon RI-mediated signal transduction. *Biochem J* **312** (Pt 1): 145–150

Zanke BW, Boudreau K, Rubie E, Winnett E, Tibbles LA, Zon L, Kyriakis J, Liu FF and Woodgett JR (1996) The stress-activated protein kinase pathway mediates cell death following injury induced by cis-platinum, UV irradiation or heat. *Curr Biol* **6**: 606–613

Zha J, Harada H, Yang E, Jockel J and Korsmeyer SJ (1996) Serine phosphorylation of death agonist BAD in response to survival factor results in binding to 14-3-3 not BCL-$X_L$. *Cell* **87**: 619–628

*© 2001 Cancer Research Campaign*
